# FTIR as a Method for Qualitative Assessment of Solid Samples in Geochemical Research: A Review

**DOI:** 10.3390/molecules27248846

**Published:** 2022-12-13

**Authors:** Yana Tkachenko, Przemysław Niedzielski

**Affiliations:** Department of Analytical Chemistry, Faculty of Chemistry, Adam Mickiewicz University, Uniwersytetu Poznańskiego 8, 61-614 Poznań, Poland

**Keywords:** FTIR, ATR, DRIFT, 2D-IR, SR-FTIR, geological samples, soil mineral composition, sediments

## Abstract

This study aims to collect information about soil investigation by FTIR. As we know, the FTIR technique is most often used in organic and bioorganic chemistry, while in geochemistry FTIR spectroscopy is not used very often. Therefore, there is a problem with the identification and interpretation of the IR spectra of minerals contained in sediments and soils. The reason for this is a deficiency of data about characteristic wavenumbers for minerals. Therefore, this study reviews and sums up, in one place, published articles that are connected to an investigation of minerals from 2002 to 2021 (based on the Scopus database). Additionally, the present review highlights various analytical techniques (ATR-FTIR, DRIFT, 2D-IR, and SR-FTIR) and discusses some of them for geochemical study. Additionally, the study describes helpful tools in the data pre-processing of IR spectra (normalization, baseline correction, and spectral derivatives).

## 1. Introduction

Geochemistry is the discipline of the earth sciences which studies the composition and distribution of chemical elements, isotopes, compounds, and minerals in the natural environment [1] using analytical techniques. The main objects of investigation in geochemistry most often are soils and sediments, which mainly have various natural elements consisting of inorganic and organic components [2].

There are lots of different analytical techniques which are helpful in the chemical characterization of soils. Basically, these are highly accurate instrumental techniques for elemental analysis such as Atomic Absorption Spectroscopy (AAS), Inductively Coupled Plasma Optical Emission Spectroscopy (ICP-OES), Inductively Coupled Plasma Mass Spectroscopy (ICP-MS), and others that require complex sample preparation and transformation sample from a solid state into a liquid [3,4,5].

Analytical chemistry has techniques which have facilities for the analysis of solid samples without difficult sample preparation, which is more convenient for chemists. For example, X-Ray Fluorescence Spectroscopy (XRF) is used for elemental analysis and Fourier Transform InfraRed Spectroscopy (FTIR) [6,7] is used to define the vibrational deformations of chemical bounds.

It is known that FTIR spectroscopy is a helpful analytical tool for organic chemistry and biochemistry researchers. It can identify unknown materials and determine the quality and/or the number of components in a sample. The advantage of this analytical technique is that it can be used for sample analysis in different states such as liquids, solids, pastes, powders, films, and also gases [8]. This method does not require a huge amount of samples and is characterized by quick and easy sample preparation and a short analysis time. Moreover, its principle of work is very simple. Additionally, it can provide even mineralogical information [9] that is the best for our knowledge, because we can investigate the efficiency of ATR-FTIR spectroscopy for qualitative analysis of mineral mixtures in natural samples (e.g., postglacial deposits).

Nowadays, there are 1730 literature references (reviews) with the keyword “FTIR”, 155 with “ATR-FTIR”, and only 30 with “ATR-FTIR, soil” in the Scopus database. Therefore, this review will collect data about sedimentary studies by FTIR using ATR and DRIFT accessories. Additionally, will collect characteristic wavenumbers for inorganic compounds/minerals in one place, adding novelty to this review.

## 2. Construction and Working Principle

FTIR begins its development with the invention of the interferometer by Michelson in the 1880s. The detailed history of Fourier Transform InfraRed Spectroscopy can be found in [10,11,12,13,14].

This technique is an integral part of IR spectroscopy and, therefore, the principle of its work is similar to dispersive spectroscopic techniques. An excellent FTIR theory and principle of working is provided by Smith [15]. The construction of the Fourier Transform InfraRed Spectrometer also contains almost all other spectrometer’s next components: An energy source, a sample compartment, and a detector that directs signals to the computer. However, there is one thing that sets it apart from all others: it is a Michelson interferometer. It consists of three active components: two mirrors (a fixed and a moving or scanning) and a beam splitter [10,16,17,18].

According to [9,10,11,16,17,18,19,20,21,22,23], in an interferometer, the beam of IR radiation falls on a beam splitter that divides it into two beams with approximately equal intensities. One-half of the light passes through the beam splitter and is reflected by the stationary mirror back to the beam splitter while the other is falling on the scanning mirror and, in the same way, reflects back to the beam splitter. The two reflected beams from mirrors are recombined, creating interference, and then directed to a detector. As a result, we acquire a signal called an interferogram [16,19,20,21], which is recoded by a special mathematical formula within the computer program.

The advantages and disadvantages of interferometry are detailed in [10,23,24]. Additionally, the advantages and disadvantages of FTIR over other IR can be viewed in more detail in [15,24,25] and some information is presented in Table 1.

## 3. Sampling Techniques, According to the Research Paper

Due to the fast development of analytic techniques, many new sampling techniques appeared. Among them, we can distinguish attenuated total reflectance, diffuse reflectance, transmittance, photoacoustic spectroscopy, and microspectroscopy [26]. Additionally, they more commonly occur in biochemistry and, in analytical chemistry, more modern IR techniques, such as two-dimensional infrared spectroscopy and synchrotron radiation-based FTIR spectromicroscopy are used.

Two-dimensional infrared spectroscopy (2D-IR) is a recent novel technique and has been rapidly developing since 1998 [27]. According to Ghosh et al. [28], this technique can convey information on molecular systems such as homogeneous and inhomogeneous spectral broadening effects, vibrational anharmonicity, spectral diffusion, energy relaxation, chemical exchange, and conformational interconversions. Contradictory to traditional FTIR, the method of 2D-IR allows us to obtain greater structural detail due to the presence of cross peaks, which help clear up the information hidden in crowded bands and interpret information on the 3D structure [29]. More detail about the principle of its work can be viewed in [27,28,29,30,31,32,33,34].

In fact, 2D-IR spectroscopy is more popular in biochemical research, especially in the investigation of proteins, peptides, and DNA. Unfortunately, there were no articles found where this technique was used in geochemical studies.

Synchrotron radiation-based on infrared spectromicroscopy (SR-FTIR) is an analytical technique with high resolution that allows the studying of chemical and biological (on a cellular level) processes in the micrometer scale and with a time resolution in the range of seconds to minutes. According to Yu and Holman [35,36], this method has features of three existing technologies, namely (1) conventional mid-IR spectroscopy, where vibrational spectra are molecular characteristics, (2) using microscopy to visually detect interesting points and then directing an IR beam to them, and (3) a high S/N ratio due to the extremely bright infrared source based on synchrotron radiation. SR-FTIR spectromicroscopy is a fast, direct, and nondestructive analytical approach which can be used in environmental, geological, and geochemical research that allow us to improve our understanding of organomineral interactions and C sequestration in soil [37].

The information about the principle of the synchrotron radiation-based Fourier Transform InfraRed analytical technique, how this approach is used in geochemical and in other studies, and also the preparation ways of the samples, has been reported [35,36,37,38,39,40,41,42,43,44,45,46].

Attenuated total reflection (ATR), also called internal reflection spectroscopy (IRS) [22] is often used for analysis as easily and nondestructive technique and has been used mainly for surface analysis and analysis of bulk materials [47]. Each ATR accessory consists of a high refractive index (HRI) material such as diamonds, zinc selenide (ZnSe), or germanium [9]. When a beam of IR radiation travels on HRI material, the light is many times internally reflected. This phenomenon creates an evanescent wave. At the moment when a sample makes contact with an evanescent wave, the last one is absorbed by the sample and, consequently, the evanescent wave will be attenuated. The attenuated beam reflects the HRI material and after that is directed to the detector [8,22]. At the time of contact, the beam of radiation penetrates just a few micrometers (~0.1–5) into the sample. Thus, the depth of penetration is proportional to the wavelength and the intensity of the absorbed radiation is dependent on the amount of the sample in contact with the surface of the ATR element [10,22]. One more very important parameter which needs to be controlled is the contact area between the sample and the HRI crystal. In the case of geological samples, the particle size allocation is the main product characteristic that has to be controlled [17]. For this reason, grinding and multiple measurements of a mixed sample each time seems advisable.

According to Smith [47], prisms and accessories for ATR spectroscopy can be divided into two groups: A single-reflection type with a hemicylinder crystal and a multiple-reflection type with a trapezoidal crystal. The possibility to control the ATR signal intensity and changing the size of solid samples make the second type of ATR prism more popular than single reflection setups. More information about horizontal and chamber sampling accessories can be found in [47].

DRIFT (Diffuse Reflectance Infrared Fourier Transform) spectroscopy is another type of device, which is used in geochemical investigation. The basic principle of its work is measuring reflected light or, in other words, diffuse-reflected light, which is obtained as a result of partially absorbing light by a sample and then diffusely re-emitting this light [14,47,48,49].

In [14,47,50,51,52,53], the authors have described, in detail with equations, the principle of DRIFT working with the Kubelka–Munk function.

Other sampling techniques can be viewed in [10,11,26,47].

## 4. Sample Preparation for ATR and DRIFT Accessories, According to the Research Paper

Sample preparation is an important part of analytical study even if an analytical technique does not require it, such as FTIR. Many scientific works, which are connected with an investigation on FTIR, are describing the process of sample preparation in the same way. All of the viewed articles accented that, in the process of sample preparation for FTIR, it is very important to be a dried and finely ground sample because wetness and particle size strongly influence the resolution of the spectrum. The next step is mixing the sample with KBr or KCl powder. This process is used for DRIFT and ATR techniques. Although, Aleksashkin et al. [54] accented on the inexpedient mixing of soil samples with potassium bromide. According to their opinion, compounds that are included in soils may be chemically reacted with KBr or KCl. Additionally, at the moment of pressing the tablet, there is a possibility of changing the structure of crystalline substances and, as a result, proving the incorrectness of the obtained spectra and interpreted results.

For published papers [55,56], which studied inorganic phosphates and clay minerals, sample preparation was characterized similarly to the above-mentioned articles. The authors of this study, similar to [54], did not use KBr for samples that were analyzed by an ATR unit.

Soriano-Disla et al. [57] investigated agricultural and grazing European soils using DRIFT. Due to this, the authors prepared 60 samples by sieving to <2 mm that were oven dried at 40 °C for 12 h. MIR spectra were obtained in the range of 4000–500 cm^−1^ at a resolution of 8 cm^−1^. The final spectrum of a sample is the average of approximately 60 scans. Salama et al. [58] studied iron ores in different geological environments and prepared samples for analysis by mixing a 1–2 mg sample with 200 mg of potassium bromide and then pressing it into pellets. Margenot et al. have described, in detail, soil preparation for analysis by DRIFT. The authors recommended the dilution of soil samples using KBr (2–10% sample) [59]. Crystal–chemical features of glauconite were investigated by Simakova et al. [60]. Before FTIR analysis, samples were prepared by pressing tablets, which contain 0.8 g KBr and some amount of the finely ground sample. Every spectrum was obtained in the MIR range of 4000–400 cm^−1^ at a resolution of 2 cm^−1^ over 256 scans.

## 5. Shifts and Overlapping in FTIR Spectra

Working with an IR spectrum of minerals requires understanding several factors which influence the position of characteristic absorption bands. Povarennykh focuses in [61] on factors that affect the shift of bands such as the valencies of cation and anion, the coordination number of cation, the coefficient of relative bond strength, which varies from 1 to 2 according to the degree of covalency of the bond, interatomic distance cation–anion, and the reduced mass of cation. According to [61], increasing a cation valency in the range from 1 to 6 and decreasing the coordination number shifts the absorption band to higher frequencies. On the other hand, increasing atomic mass leads to decreasing frequency. More detailed information with diagrams and tables can be viewed in [61].

During the spectral analysis of sediments, there often occurs a poorly resolved spectrum due to the presence of an overlapping effect. The location of overlapped peaks is found by derivative spectroscopy [15,62,63,64] which involves the analysis of the second or the fourth derivative of the spectrum [62,63]. Additionally, the authors in [62] note that occur another method where the measured infrared spectrum is divided by the instrumental function to change signal deformation. However, they more attentively studied the second and fourth derivatives with detailed calculations and different models. As a result, a procedure described by the authors of [62] allowed for the determination of the number and the locations of overlapped peaks. Spectral derivatives are a technique that belongs to data pre-processing and will be viewed in detail in Section 6.

## 6. Data Pre-Processing

Data pre-processing is an important part of spectral data analysis. According to [65], the main goals of pre-processing are as follows: (1) improving the robustness and accuracy of subsequent quantitative or qualitative analyses; (2) the possibility to transform raw data into a more understandable format; (3) the detection and elimination of outliers and trends; (4) the devaluation of the scale of the data mining task; and (5) the possibility to select features for the elimination of irrelevant and unnecessary information.

Pre-processing procedures can be divided into eight different categories, namely exclusion (cleaning), normalization, filtering, detrending, transformations, feature selection, folding or unfolding, and other methods [66]. The choices of every procedure depend on the analysis goal, the physical state of the sample, and the time and computing power available [65]. This review will examine the most widely used techniques, such as normalization, baseline correction methods, and spectral derivatives. More detailed information about other pre-processing techniques can be found in [63,65,66,67,68].

### 6.1. Normalization

Normalization as a pre-processing technique is used to scale the spectra within a similar range. The work with IR spectra follows types of normalization, such as Min-Max normalization, 1-norm, and vector normalization [66].

Min-Max normalization is the simplest normalization method. During this procedure, spectra are first offset-corrected by setting the minimum intensity of the whole spectrum and then scaled with a maximum intensity value equaling one [66].

The main goal of 1-norm normalization is mean centering; that is, the average spectral intensity is subtracted from the spectrum. Next, mean-centered spectra are scaled in a way that the sum of the absolute values of all intensities equals one [66].

Vector normalization or 2-norm base is the process of dividing a mean-centered spectrum by the square root of the sum of the mean-centered intensities squared [66].

### 6.2. Baseline Correction

In IR spectroscopy, spectral baselines can be distorted as a result of scattering or absorption by the supporting substrate [66]. Chukanov notes in [68] two kinds of algorithms that are used for baseline correction. Using the first algorithm allows the drawing of curves in a manner that permits full control of the line curvatures by the user. This procedure can be based on polynomial methods of using least-squared fitted lines or the method of Bezier. The other one is a fast automatic algorithm based on repeated curve fitting where orthogonal polynomials are used [68]. These kinds of algorithms and other algorithms are described in detail in [66].

### 6.3. Spectral Derivatives

According to Section 5, spectral derivatives are a useful tool for increasing the resolution of infrared spectra. Moreover, Lasch [66] focuses on another advantage of this type of pre-processing technique and, namely, the minimization of the contributions of baseline offsets or slopes. Thus, the intricacy of spectra is reduced, which facilitates the spectral curve fitting by reducing the number of fit parameters.

Among spectral derivatives, the most popular smoothing/derivative method is the Savitzky–Golay method [66,67]. According to [66,67], the main concept of the Savitzky–Golay method is the idea of a convolute and a convolution function. More detail about this method and also another derivation method, the so-called Norris–Williams derivation, that can be viewed in [66,67].

## 7. Identification of IR Spectra for Geological Samples

Identification of an IR spectrum of geological samples is a complicated process because sediments are a mixture of different minerals and peaks and the fingerprint zone of the spectrum can overlap. The most common minerals, which occur in the soils, are different kinds of clays (for example, kaolinite), calcites, different morphological kinds of silica, etc.

During an investigation of sediments, the IR spectrum can be conditionally divided into two zones. The first zone over frequency ranges from 3800 to 3500 cm^−1^ responsible for the OH stretching vibration and another one in the range from 1120 to 400 shows structural deformation of the Si-O group and other characteristic groups (fingerprint zone) [56]. In the case of soils should not be forgotten organic matters, so-called “humic substances”. These organic matters are usually distributed in the 2925 cm^−1^ for lipids and in the wavenumbers range 1750–1200 cm^−1^ for lignins and proteins. Parikh et al. [11] excellently described and collected the major characteristic wavenumbers not only for organic matters but also for bacteria and biomolecules, which are presented in the natural soils.

Table 2 of this study sums up the main information about FTIR analysis and interpretation of characteristic wavenumbers for inorganic components of natural solid samples.

### 7.1. Vibration of (OH)-Group in the Sediments/Soils

**Figure 1:**
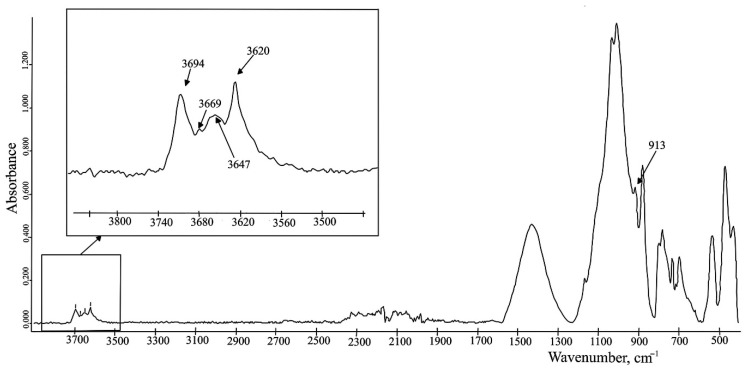
The FTIR spectrum of a postglacial deposit (Petuniabukta, central Spitsbergen (Svalbard)) which contains kaolinite. The spectrum was obtained by the authors of this review.

**Figure 2:**
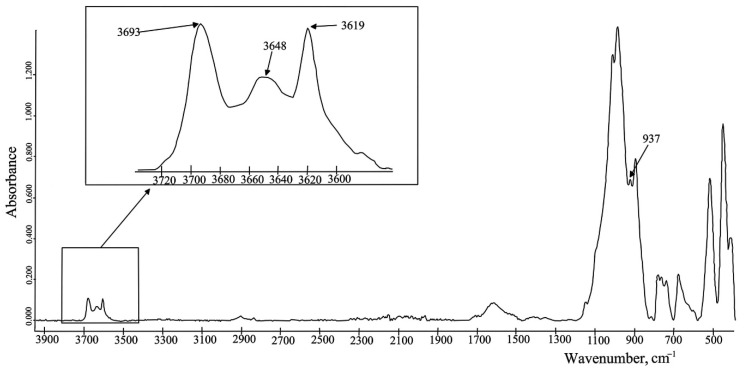
The FTIR spectrum of a postglacial deposit (Petuniabukta, central Spitsbergen (Svalbard)) which contains dickite. The spectrum was obtained by the authors of this review.

According to Madejova [56], the stretching vibration (OH)-group in the soil plays an important role in the interpretation of minerals. Considering the amount and size of absorption peaks of OH in the wavenumbers range 3800–3500 cm^−1^, there can easily be indicated clays. As shown in Figure 1, kaolinite has four absorption bonds in the OH stretching region: two strong bands at 3694 and 3620 cm^−1^ and two weak peaks at 3669 and 3647 cm^−1^ [56]. The same results were obtained by Soriano-Disla et al. [57], Aleksashkin et al. [54], And Oloyede et al. [69]. One more absorption band at 914 cm^−1^ is an Al_2_OH bending vibration and is also characterized by kaolinite [54,56]. An investigation in [56] also showed that dickite is spectrally similar to kaolinite (Figure 2). The stretching peaks and the bending bands of the (OH)-groups for dickite are at 3703, 3653, 3620 cm^−1,^ and 936 cm^−1^, respectively [56,57].

Madejová [56] claimed that dioctahedral smectites such as montmorillonite, nontronite, hectorite, and saponite, have only a single band in the OH stretching region. Characteristic frequencies for them are presented in Table 1.

According to Simakova [60], who studied glauconite, highlighted that following the stretching band of (OH)-groups appears at 3534.4 cm^−1^ for the bonds Fe^3+^OHFe^3+^, at 3543.2 cm^−1^ for the bonds MgOHFe^2+^, at 3558.4 cm^−1^ for the bonds MgOHFe^3+^/or AlOHFe^2+^, at 3566.2 cm^−1^ for the bonds AlOHFe^3+^, at 3583.9 cm^−1^ for the bonds MgOHMg, at 3604.8 cm^−1^ for the bonds AlOHMg and, for the bonds AlOHAl, at 3619.0 and 3647.3 cm^−1^. The bending deformation of the (Mg)Fe^3+^OHFe^3+^ in the glauconite was observed at 818 cm^−1^.

Salama et al. [58] investigated iron ores. Goethite, which occurred in the iron ores, has three vibrational modes of the (OH)-groups. One of them is stretching bands at 3416 and 3133 cm^−1^. Two others are bending bands at 892 and 795 cm^−1^.

### 7.2. Vibrational Deformations of Si-O in the Sediments/Soils 

Vibrational deformations of the Si-O in the soils are useful for identifying layer silicates. According to [56,59], clays have different constructions within the layers that influence the location of the bands in the spectrum. The 1:1-layer silicates have three Si-O absorbance peaks in a frequency range from 1120 to 950 cm^−1^ (kaolinite and dickite), while the 2:1-layer silicates have a single broad band in the wavenumber 1030–1010 cm^−1^ range.

Glauconite absorbs at 1121, 1077, 1026, 992, and 957 cm^−1^, which corresponds Si-O-Si stretching deformation at 914 cm^−1^ (stretching Si-O-Al), and at 460 cm^−1^ of the banding deformation Si-O-Si(Al) [54,60].

Smectites, such as montmorillonite and nontronite, have one broad Si-O stretching band at 1030 and 1019 cm^−1^, respectively, while hectorite and saponite absorb at 1012 and 1009 cm^−1^, respectively [54,56].

The spectra of soils of the montmorillonite–kaolinite type show Si-O bending absorptions at 694 or 693 cm^−1^, in the 527–514 cm^−1^ range, and also in the 427–416 cm^−1^ region. Spectra of these kinds of soils have also in the 668-645 cm^−1^ region the deformation band Si-O(-Si) and a bending absorption Si-O-(Si,Al,Mg) in the frequency range from 464 to 447 cm^−1^ [54].

According to published papers [3,54,58,69,70], stretching bands Si-O within quartz can be found at 1082, 798, and 779 cm^−1^.

### 7.3. Carbonates, Nitrates, Sulphates, and Phosphates in the Sediments/Soils

Carbonates are common components in geological samples and occur in sedimentary rocks, which are called limestone. Among the carbonates, the most abundant minerals are calcite (CaCO_3_) and dolomite (MgCa(CO_3_)_2_) [71]. According to [3,54,55,70,71], characteristic frequency vibrations for dolomite were indicated at 1814, 880, and 730 cm^−1^, while the calcite was identified with wavenumbers at 1796, 875, and 712 cm^−1^. The wavenumber at 875 cm^−1^ is a symmetric banding deformation of the C-O-C within the carbonate ion. An asymmetric banding vibration of the C-O-C in the calcite occurs in the 717–712 cm^−1^ range. Both of the minerals have a stretching broad band in the 1425∓1393 cm^−1^ region.

The FTIR spectrum of soils is complicated and bands of nitrates, sulfates, and phosphates are difficult to find on the spectrum due to overlapping more intensive signals on their weak signals. Salama et al. [58] identified nitrate bands at 1796 and 1384 cm^−1^, and also sulfate bands at 982 and around 610 cm^−1^.

Campos et al. [55] identified phosphate bands between the 1200–984 cm^−1^ and 634–450 cm^−1^ regions. In this article, we described two types of vibrations for phosphate ions. There are bending deformational vibrations of the O-P-O and asymmetric stretching vibrations of the P-O bond. The last one was indicated in the spectral range from 1182 to 1005 cm^−1^. The bands of deformation vibration of the O-P-O fragment were observed in the 634–539 cm^−1^ and 516–451 cm^−1^ regions.

### 7.4. Water and Carbon Dioxide in the FTIR Spectrum

**Figure 3:**
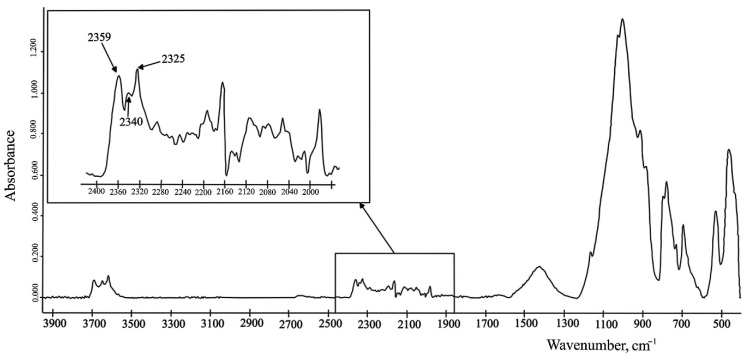
The level of noise on the spectrum of a postglacial deposit (Petuniabukta, central Spitsbergen (Svalbard)). The spectrum was obtained by the authors of this review.

Water vapor and carbon dioxide are always in the atmosphere and the ATR-FTIR spectrum of soils always shows noise levels in the region of 2500–2000 cm^−1^ (Figure 3). The frequency range from 2400 to 2300 cm^−1^ shows peaks of carbon dioxide [70]. Shi et al. [72] observed on the spectrum peaks at ca. 2361 and ca. 2336 cm^−1^ what was a stretching mode of the CO_2_ and another absorption band at ~667 cm^−1^ (banding vibration of the CO_2_). Water deformational vibrations were found in [72], at ~3385 for stretching vibrations and around 1635 cm^−1^ for banding deformations of the H-O-H, respectively.

### 7.5. Organic Matters in the Soils

The natural soils, except for minerals, also include many organic matters. Soil organic matter (SOM) is heterogeneous material comprising matter of plant and animal origin at different degrees of degradation [73]. According to Parikh et al. [11] and to Stenberg et al. [74], organic molecule bonds such as C-O, C=O, C-C, C-H, O-H, N-H, N=C, and S-H, which are present in SOM, absorb IR radiation in the MIR region and their overtones and combination bands occur in the vis-NIR.

**Figure 4:**
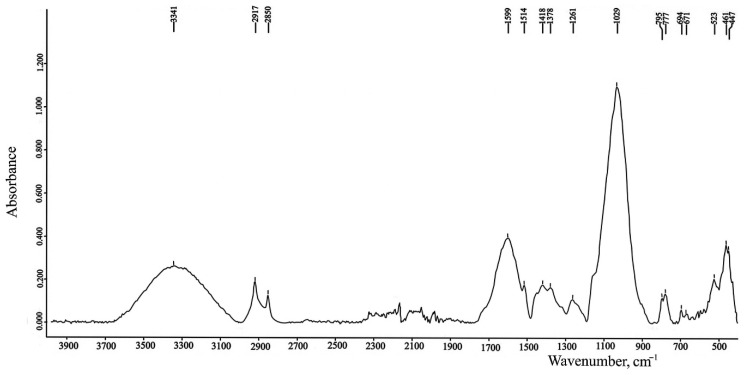
The ATR-FTIR spectrum of soil (Petuniabukta, central Spitsbergen (Svalbard)) which contains SOM. The spectrum was obtained by the authors of this review.

For alcohol and amines, stretching deformation of the OH or NH bond occurs in the frequency region from 3700 cm^−1^ to 3000 cm^−1^. Dutta [18], notices that the OH absorptions in contrast to NH stretchers are generally quite intense and smoothly curved (Figure 4).

Nuzzo et al. [75] and Krivoshein et al. [76] indicated C-H stretching of CH_2_ groups at 2920 cm^−1^ (asymmetric) and 2850 cm^−1^ (symmetric), which are usually attributed to SOM (Figure 4). According to [76], C-H aromatic bending appears at 2030 cm^−1^ in the frequency range of 1783–2000 cm^−1^, which can be observed C=O stretching vibration and N-H bending deformation, and -C=C- stretching can be founded at the wavenumber region between 1680 and 1710 cm^−1^.

Krivoshein et al. [76] claimed that bands in the wavenumbers ranging between 1650–1580 cm^−1^ and 1450–1220 cm^−1^ are primarily associated with soil water and silicon dioxide and bending vibrations in quartz and hydrosilicates, respectively. According to the authors’ opinion, these regions cannot be used for SOM quantification.

Stenberg et al. [74] described band assignments for fundamental MIR absorptions of soil organic matter with their overtones and combinations in the NIR region. According to this, in the MIR region, wavenumbers in the SOM are the following: for aromatics, compounds stretching vibration C-H at 3030 cm^−1^ (vis-NIR: 1650, 1100, and 825 nm); for amine, N-H stretching bands and bending deformation at 3330 cm^−1^ and 1610 cm^−1^, respectively (vis-NIR: 2060, 1500, 1000, and 751 nm); for alkyl, asymmetric and symmetric stretching bands at 2930 cm^−1^ and 2850 cm^−1^, respectively (vis-NIR: 1650, 1706, 1754, 1138, 1170, 853, and 877 nm); the stretching vibration of C=O for carboxylic acids and amides at 1725 cm^−1^ and 1640 cm^−1^, respectively (vis-NIR: 1930 and 1449 nm, and 2033 and 1524 nm, respectively); for aliphatic compounds, C-H stretching bands appear at 1465 cm^−1^ (vis-NIR: 2275 and 1706 nm); and for stretching deformation of C-H in the methyl occurring in the 1445–1350 cm^−1^ range (vis-NIR: 2307–2469 and 1730–1852 nm).

## 8. Conclusions

FTIR spectroscopy is a useful and non-destructive technique that does not require difficult sample preparation and using aggressive organic solvents. Using special accessories, such as ATR and DRIFT, can provide analysis to maximum sensitivity. Due to this, it can be distinguished different kinds of minerals which contain in the samples of the soils. ATR-FTIR and DRIFT have low noise levels for an inorganic matrix that is very useful for qualitative analysis in geochemistry.

New perspectives in the detailed characterization of minerals provide opportunities to combine various FTIR techniques with microscopic systems. In addition, spectral libraries implemented by manufacturers allow for a significant simplification and, partially, automation of the process of identifying minerals. Currently, the FTIR technique is not a complete alternative to other mineral identification procedures, but it is a useful auxiliary tool. Working with FTIR does not restrict only the use of ATR and DRIFT accessories, as long as there exist more modern techniques, such as 2D-IR and SR-FTIR. However, these new methods are barely used in geochemical research. By taking into consideration their potential, 2D-IR and SR-FTIR might play a crucial role in achieving more detailed data.

Presented in this review is a collection of characteristic wavenumbers and a description of the most widely used data and pre-processing techniques that will help interpret the IR spectra of geochemical samples such as sediments, minerals, and soils.

## Figures and Tables

**Table 1:** Advantages and disadvantages of FTIR over other IR spectrometers, according to [10,15,23,24,25].

Categories	FTIR	Other IR
SNR or Jacquinot’s Advantage	Has a high throughput and, as a result, a high SNR.	IR beams pass through prisms, slits, and gratings that restrict the intensity of the beam and reduce throughput.
Multiplex or Fellgett’s Advantage	Simultaneously encode all wavelengths of light.	Every wavelength must be measured individually.
Laser-Referenced Frequencies or Conne’s Advantage	Uses a He-Ne laser as an interferometer reference. This allows the wavenumbers in a measured FTIR spectrum to be determined with a precision of ±0.01 cm^−1^.	Needs to be calibrated by the users.
Artifacts	The major disadvantage of FTIR. These are features present in the spectrum of a sample that is not from the sample (peaks of water vapor and carbon dioxide).	There are no extraneous peaks in the spectrum of the sample.

**Table 2:** Characteristic wavenumbers and parameters of measurement for samples of sediments, according to papers published from 2002 to 2021.

Matrix Sample Type	Accessories	Parameters of Measurement	Sample Preparation		Characteristic Wavenumbers, cm^−1^		Ref.
			Reagents	Other	Peaks	Description	
Geological samples of rocks and sediments (limestone, sandstone, and sediments)	ATR	MIR range: 4000–400 cm^−1^; scanning: 32; resolution: 4 cm^−1^	K_3_[Fe(CN)_6_]; KBr	ND ^c^	798 and 779	silica	[3]
					875 and 712	calcium carbonate	
Clay mineral	ATR; DRIFT	MIR range: 4000–400 cm^−1^; scanning: 128; resolution: 4 cm^−1^	without any dilution in KBr	ND	3620, 3653, 3669, and 3695	ν-OH for kaolinite	[36]
					3620, 3653, and 3703	ν-OH for dickite	
					3648 and 3694	ν-OH for chrysotile	
					3620	ν-OH for montmorillonite	
					3567	ν-OH for nontronites (FeFeOH)	
					~3680	ν-OH for hectorite and saponite	
					~3430	H-O-H vibration into all smectites	
					1120–1000	ν-Si-O for kaolinite and dickite	
					960	main ν-Si-O for chrysotile	
					1030	ν-Si-O for montmorillonite	
					1019	ν-Si-O for nontronite	
					1012	ν-Si-O for hectorite	
					1009	ν-Si-O for saponite	
					914	δ-OH for kaolinite (Al_2_OH)	
					936	δ-OH for dickite (Al_2_OH)	
					~600	δ-OH for chrysotile (Mg_3_OH)	
					916	δ-OH for montmorillonite (Al_2_OH)	
					844	δ-OH for montmorillonite (AlMgOH)	
					817 676	δ-OH for nontronite (Fe_2_OH) Fe-O out-of-plane vibration	
					655	δ-OH for hectorite (Mg_3_OH)	
					660	δ-OH for saponite (Mg_3_OH)	
Agricultural and grazing European soils	DRIFT	MIR range: 4000–500 cm^−1^, scanning: 60; resolution: 8 cm^−1^, d ^d^ = 9 mm	ND	sieved: <2 mm; dried: 40 °C for 12 h	3632 and 1630;	illites and/or smectites	[37]
					2000–1800 and 1180–1160 and 1044	quartz	
					3696 and 3628	kaolinite	
					670, 740, 780, and 860	Ti oxide	
Iron ore	ND	MIR range: 4000–400	KBr	Mixing of a 1–2 mg sample with 200 mg KBr; pressed	3416 and 3133	ν ^a^-OH for goethite	[38]
					1620 and 1634	H_2_O	
					982 and ~610	SO_4_ band	
					1384 and 1796	NO_3_ band	
					1085	quartz or polysaccharide carbohydrates	
					892 and 795	δ ^b^-OH and γ ^b^-OH for goethite, respectively	
					418, 455, and 670	other absorption bands of goethite	
					471 and 540 or 545	hematite	
					1082	Si-O in the quartz	
					1032, 1011, 940, and 914	Si-O-Si, Si-O-Al, Al-O-H for kaolinite respectively	
					540 471	Si-O-Al Si-O common in the kaolinite and hematite	
Minerals and SOM ^e^	ATR and DRIFT	ND	KBr	finely ground <900 μm for DRIFT;	3750–3400	ν-OH for phyllosilicates	[38]
					950–600	δ-OH for phyllosilicates	
					1200–700 and 700–400	ν-Si-O	
					1120–950	Si-O for 1:1-layer silicates	
					1030–1010	Si-O for 2:1-layer silicates	
					3800–2800	ν-OH for allophane and imogolite	
					1700–1550	δ-OH for allophane and imogolite	
					1200–800	ν-Si-O for allophane and imogolite	
Glauconite	ND	MIR range: 4000–400 cm^−1^; scanning: 256; resolution: 2 cm^−1^	KBr	ND	1121, 1077, 1026, 992, and 957	ν-Si-O-Si	[40]
					914	ν-Si-O-Al	
					818	δ-[(Mg)Fe^3+^OHFe^3+^]	
					677	ν-[Fe^3+^-O]	
					533	ν-[Al-O]	
					497	ν-[Mg-O]	
					460	δ-Si-O-Si(Al)	
					3534.4	ν-OH for Fe^3+^OHFe^3+^	
					3543.2	ν-OH for MgOHFe^2+^	
					3558.4	ν-OH for MgOHFe^3+^ or AlOHFe^2+^	
					3566.2	ν-OH for AlOHFe^3+^	
					3583.9	ν-OH for MgOHMg	
					3604.8	ν-OH for AlOHMg	
					3619.0 and 3647.3	ν-OH for AlOHAl	
River bank, alluvial sediments, and lake bed sediments	DRIFT	MIR range: 4000–400 cm^−1^; scanning: 16; resolution: 4 cm^−1^; d = 3 mm	ND	lyophilizing	1796, 875, and 712	CO_3_ band for calcite	[43]
					1814, 880, and 730	CO_3_ band for dolomite	
CO_2_/Brine/Rock systems	ATR	MIR range: 4000–400 cm^−1^; scanning: 500 resolution: 2 cm^−1^; cell temperature 50 °C; cell pressure 0.7, 2.8, 5.5, and 8.3 MPa	ND	powdered; sieved to 73 μm; mixed with brine	2361 and 2336	ν-CO_2_	[44]
					667	δ-CO_2_	
					3385	ν-H-O-H	
					1635	δ-H-O-H	
					1300–1400	aqueous carbonate and bicarbonate ions region	
Phosphate rocks	DRIFT and ATR	MIR range: 4000–400 cm^−1^; scanning: 100; resolution: 8 cm^−1^	KBr	mixed with KBr: 10% sample/90%KBr for DRIFT	1182–1005	ν-P-O	[35]
					634–539	δ-O-P-O symmetric	
					516–451	δ-O-P-O asymmetric	
					1458–1427	ν-C-O asymmetric (into CO_3_)	
					870	δ-C-O-C symmetric (into CO_3_)	
					717 and 672	δ-C-O-C asymmetric (into CO_3_)	
					672, 588, ~470, and ~460	ν-Al_2_OH	
					~1180, ~1040 and 800	δ-Al_2_OH	
					~840	δ-Al-O-Fe	
					3045–3033	ν-N-H	
Foothill chernozems	ND	MIR range: 4000–400 cm^−1^	Not used	ground to a fine powder	3695	ν-(Al,Fe,Mg)-OH	[34]
					3627–3617	ν-Al-Al-OH	
					~915	δ-Al-Al-OH	
					3405–3357	ν-H-O-H	
					1639–1629	δ-H-O-H	
					1425–1393	ν-C-O into CaCO_3_	
					1022–1000	ν-Si-O	
					874–872 and 712	δ-C-O into CaCO_3_	
					797–794 and 779-776	ν-Si-O into SiO_2_	
					694–693 and 528–513	δ-Si-O	
					649–645	δ-Si-O(-Si)	
					564–459	δ-Si-O-(Si,Al,Mg)	
					428–413	δ-Si-O	
Bentonite Clay	ND	MIR range: 4000–650 cm^−1^; resolution: 1 cm^−1^	ND	dried; powdered; sieved to 125 μm	685.8	OH deformation	[41]
					749.2	Al-O-Si	
					777.1	Si-O deformation	
					909.5	Al-Al-OH	
					997.1	Si-O planar stretching	
					3617.4	OH-stretching	
					3690.1	structural OH stretching	
Silicate matrix soil (sod-podzolic and chernozems)	DRIFT, ATR, and FTIR-PAS	MIR range: 4000–100 cm^−1^	ND	dried; sieved	3700	unbonded SiO-H stretch (DRIFT)	[42]
					3690–3680	hydrogen-bonded SiO-H…H_2_O stretch (amorphous) (DRIFT and ATR)	
					3670–3650	ν-OH of inner-surface hydroxyl groups (DRIFT)	
					3620	ν-Al(Mg)SiO-H (DRIFT and ATR)	
					1650–1640	absorbed liquid water bend, ν-HO-H (DRIFT and ATR)	
					1460	carbonate (DRIFT and ATR)	
					1420	ν-Mg-OH (DRIFT and ATR)	
					1185	amorphous silica (DRIFT)	
					1165–1153	SiO_2_ lattice (DRIFT and ATR)	
					1115–1105	amorphous silica (DRIFT and ATR)	
					1095	SiO_2_ silicate ν-Si-O (DRIFT)	
					1080–1075	O-Si-O lattice stretch (ATR)	
					1010–995	SiO_2_ ν-Si-O lattice (DRIFT and ATR)	
					975	SiO_2_ silicate (kaolinite and illite) (DRIFT and ATR)	
					930–910	silicate and aluminosilicate (DRIFT and ATR)	
					860	Al-OH (clay minerals) (ATR)	
					840–830	Al-OH (clay minerals), smectite, illite, and AlMgOH (DRIFT)	
					813	amorphous silica and Ti-O (DRIFT)	
					796	ν-Si-O-Si (DRIFT and ATR)	
					697–696	δ-Si-O-Si (DRIFT and ATR)	
					675–650	CO_2_ (DRIFT and ATR)	
					655–650	δ-Si-O-Si and amorphous iron oxide (DRIFT and ATR)	
					645–640	Sulfate (ATR)	
					565	PO_4_ tetrahedra (DRIFT)	
					535–525	Si-O-Al deformation in kaolinite and iron oxide (ATR)	
					470	O-Si-O bend, O-Al-O, and iron oxide (ATR)	
					430–420	Si-O deformation of kaolinite, Mg-OH, and Al-OH (clay minerals) (DRIFT and ATR)	

^a^—stretching mode; ^b^—bending mode; ^c^—no data; ^d^—diameter sample cups; ^e^—soil organic matter.

## Data Availability

The data presented in this study are available upon request from the corresponding author.

## Abbreviations

AAS	Atomic Absorption Spectroscopy
ATR	Attenuated Total Reflection
DRIFT	Diffuse Reflectance InfraRed Fourier Transform spectroscopy
2D-IR	Two-dimensional InfraRed spectroscopy
FTIR	Fourier Transform InfraRed spectroscopy
HRI	High Refractive Index
ICP-MS	Inductively Coupled Plasma Mass Spectroscopy
ICP-OES	Inductively Coupled Plasma Optical Emission Spectroscopy
IR	InfraRed
IRS	Internal Reflection Spectroscopy
MIR	Mid-InfraRed
S/N ratio	Signal-to-Noise ratio
SOM	Soil Organic Matter
SR-FTIR	Synchrotron Radiation-Based Infrared spectromicroscopy
XRF	X-Ray Fluorescence spectroscopy

## References

[1] Demetriades A. (2021). Geochemical Mapping. Encyclopedia of Geology.

[2] Sangwan P., Nain T., Singal K., Hooda N., Sharma N. (2020). Soil as a tool of revelation in forensic science: A review. Anal. Methods.

[3] Reig F. (2002). FTIR quantitative analysis of calcium carbonate (calcite) and silica (quartz) mixtures using the constant ratio method. Application to geological samples. Talanta.

[4] Evans E.H., Day J.A., Fisher A., Price W.J., Smith C.M.M., Tyson J.F. (2004). Atomic spectrometry update. Advances in atomic emission, absorption and fluorescence spectrometry and related techniques. J. Anal. At. Spectrom..

[5] Bacon J.R., Butler O.T., Cairns W.R.L., Cook J.M., Davidson C.M., Cavoura O., Mertz-Kraus R. (2020). Atomic spectrometry update – a review of advances in environmental analysis. J. Anal. At. Spectrom..

[6] Chen Z.W., Gibson W.M., Huang H. (2008). High Definition X-Ray Fluorescence: Principles and Techniques. X-Ray Opt. Instrum..

[7] Xu J.-L., Thomas K.V., Luo Z., Gowen A.A. (2019). FTIR and Raman imaging for microplastics analysis: State of the art, challenges and prospects. TrAC Trends Anal. Chem..

[8] Nandiyanto A.B.D., Oktiani R., Ragadhita R. (2019). How to Read and Interpret FTIR Spectroscope of Organic Material. Indones. J. Sci. Technol..

[9] Mohamed M.A., Jaafar J., Ismail A.F., Othman M.H.D., Rahman M.A. (2017). Fourier Transform Infrared (FTIR) Spectroscopy. Membrane Characterization.

[10] Subramanian A., Rodriguez-Saona L. (2009). Fourier Transform Infrared (FTIR) Spectroscopy. Infrared Spectroscopy for Food Quality Analysis and Control.

[11] Parikh S.J., Goyne K.W., Margenot A.J., Mukome F.N.D., Calderón F.J. (2014). Soil Chemical Insights Provided through Vibrational Spectroscopy. Advances in Agronomy.

[12] Bell R.J. (1972). Historical Sketch and Crucial Ideas. Introductory Fourier Transform Spectroscopy.

[13] (2001). Chapter 6 Instrumentation. Comprehensive Analytical Chemistry.

[14] Chalmers J.M., Griffiths P.R. (2001). Handbook of Vibrational Spectroscopy: Chalmers Vibrat 5V Set.

[15] Smith B.C. (2011). Fundamentals of Fourier Transform Infrared Spectroscopy.

[16] Settle F.A. (1997). Handbook of Instrumental Techniques for Analytical Chemistry.

[17] Reh C. (2001). In-line and off-line FTIR measurements. Instrumentation and Sensors for the Food Industry.

[18] Dutta A. (2017). Fourier Transform Infrared Spectroscopy. Spectroscopic Methods for Nanomaterials Characterization.

[19] Shaw R.A., Mantsch H.H. (1999). Near-IR Spectrometers. Encyclopedia of Spectroscopy and Spectrometry.

[20] Spragg R.A. (1999). IR Spectrometers. Encyclopedia of Spectroscopy and Spectrometry.

[21] Efimova A.I., Zaitsev V.B., Boldyrev N.Y., Kashkarov P.K. (2008). Infrared Fourier Spectrometry.

[22] Derrick M.R., Stulik D., Landry J.M. (1999). Infrared Spectroscopy in Conservation Science.

[23] Larrabee J.A., Choi S. (1993). Fourier transform infrared spectroscopy. Methods in Enzymology.

[24] Jaleh B., Fakhri P. (2016). Infrared and Fourier transform infrared spectroscopy for nanofillers and their nanocomposites. Spectroscopy of Polymer Nanocomposites.

[25] Withrow J. (2012). Infrared Spectroscopy.

[26] Hind A.R., Bhargava S.K., McKinnon A. (2001). At the solid/liquid interface: FTIR/ATR–the tool of choice. Adv. Colloid Interface Sci..

[27] Buchanan L.E., Xiong W. (2018). Two-Dimensional Infrared (2D IR) Spectroscopy. Encyclopedia of Modern Optics.

[28] Ghosh D., Deshmukh S., Chatterjee S., Sakpal S., Haldar T., Dhakad A., Kashid S., Bagchi S., Singh D.K., Pradhan M., Materny A. (2021). Two Dimensional Infrared Spectroscopy: A Structure Sensitive Technique with Ultrafast Time Resolution. Modern Techniques of Spectroscopy.

[29] Hill R.E., Hunt N.T., Hirst J.D. (2013). Studying Biomacromolecules with Two-Dimensional Infrared Spectroscopy. Advances in Protein Chemistry and Structural Biology.

[30] Noda I. (1990). Two-Dimensional Infrared (2D IR) Spectroscopy: Theory and Applications. Appl. Spectrosc..

[31] Wang J. (2017). Ultrafast two-dimensional infrared spectroscopy for molecular structures and dynamics with expanding wavelength range and increasing sensitivities: From experimental and computational perspectives. Int. Rev. Phys. Chem..

[32] Ghosh A., Ostrander J.S., Zanni M.T. (2017). Watching Proteins Wiggle: Mapping Structures with Two-Dimensional Infrared Spectroscopy. Chem. Rev..

[33] Cho M. (2009). Two-Dimensional Optical Spectroscopy.

[34] Hamm P., Zanni M.T. (2011). Concepts and Methods of 2d Infrared Spectroscopy.

[35] Yu P. (2004). Application of advanced synchrotron radiation-based Fourier transform infrared (SR-FTIR) microspectroscopy to animal nutrition and feed science: A novel approach. Br. J. Nutr..

[36] Holman H.-Y.N. (2010). Synchrotron Infrared Spectromicroscopy for Studying Chemistry of Microbial Activity in Geologic Materials. Developments in Soil Science.

[37] Lehmann J., Solomon D. (2010). Organic Carbon Chemistry in Soils Observed by Synchrotron-Based Spectroscopy. Developments in Soil Science.

[38] Bantignies J.-L., Carr L., Dumas P., Miller L., Williams G.P. (1998). Applications of Infrared Microspectroscopy to Geology, Biology and Cosmetics. Synchrotron Radiat. News.

[39] Saviello D., Pouyet E., Toniolo L., Cotte M., Nevin A. (2014). Synchrotron-based FTIR microspectroscopy for the mapping of photo-oxidation and additives in acrylonitrile–butadiene–styrene model samples and historical objects. Anal. Chim. Acta.

[40] Jungman E., Laugel C., Rutledge D.N., Dumas P., Baillet-Guffroy A. (2013). Development of a percutaneous penetration predictive model by SR-FTIR. Int. J. Pharm..

[41] Reuben S., Banas K., Banas A., Swarup S. (2014). Combination of synchrotron radiation-based Fourier transforms infrared microspectroscopy and confocal laser scanning microscopy to understand spatial heterogeneity in aquatic multispecies biofilms. Water Res..

[42] Nimer R., Kamel G., Obeidat M.A., Dahabiyeh L.A. (2022). Investigating the molecular structure of plasma in type 2 diabetes mellitus and diabetic nephropathy by synchrotron Fourier-transform infrared microspectroscopy. Spectrochim. Acta Part A Mol. Biomol. Spectrosc..

[43] Barón-Sola Á., Toledo-Basantes M., Arana-Gandía M., Martínez F., Ortega-Villasante C., Dučić T., Yousef I., Hernández L.E. (2021). Synchrotron Radiation-Fourier Transformed Infrared microspectroscopy (μSR-FTIR) reveals multiple metabolism alterations in microalgae induced by cadmium and mercury. J. Hazard. Mater..

[44] Martin M.C., Schade U., Lerch P., Dumas P. (2010). Recent applications and current trends in analytical chemistry using synchrotron-based Fourier-transform infrared microspectroscopy. TrAC Trends Anal. Chem..

[45] Holman H.N., Martin M.C. (2006). Synchrotron Radiation Infrared Spectromicroscopy: A Noninvasive Chemical Probe for Monitoring Biogeochemical Processes. Advances in Agronomy.

[46] Guilhaumou N., Sautter V., Dumas P. (2005). Synchrotron FTIR microanalysis of volatiles in melt inclusions and exsolved particles in ultramafic deep-seated garnets. Chem. Geol..

[47] (2001). Chapter 7 Sampling techniques and applications. Comprehensive Analytical Chemistry.

[48] Ferraro J.R., Basile L.J. (1985). Fourier Transform Infrared Spectroscopy: Applications to Chemical Systems.

[49] Burns D.A., Ciurczak E.W. (2007). Handbook of Near-Infrared Analysis.

[50] Simmons E.L. (1976). Reflectance spectroscopy: Application of the Kubelka-Munk theory to the rates of photoprocesses of powders. Appl. Opt..

[51] Yang L., Kruse B. (2004). Revised Kubelka–Munk theory I Theory and application. J. Opt. Soc. Am. A.

[52] Yang L., Miklavcic S.J. (2005). Revised Kubelka–Munk theory III A general theory of light propagation in scattering and absorptive media. J. Opt. Soc. Am. A.

[53] Kokhanovsky A.A. (2007). Physical interpretation and accuracy of the Kubelka–Munk theory. J. Phys. D Appl. Phys..

[54] Aleksashkin I.V., Bobra T.V., Dubas V.V. (2020). Study of the inorganic component of piedmont chernozems in the Belogorsk region of the Republic of Crimea by IR spectroscopy. Geopolit. Ecogeodynamics Reg..

[55] Campos P.V., Albuquerque A.R.L., Angélica R.S., Paz S.P.A. (2021). FTIR spectral signatures of amazon inorganic phosphates: Igneous, weathering, and biogenetic origin. Spectrochim. Acta Part A Mol. Biomol. Spectrosc..

[56] Madejová J. (2003). FTIR techniques in clay mineral studies. Vib. Spectrosc..

[57] Soriano-Disla J.M., Janik L., McLaughlin M.J., Forrester S., Kirby J.K., Reimann C. (2013). Prediction of the concentration of chemical elements extracted by aqua regia in agricultural and grazing European soils using diffuse reflectance mid-infrared spectroscopy. Appl. Geochem..

[58] Salama W., el Aref M., Gaupp R. (2015). Spectroscopic characterization of iron ores formed in different geological environments using FTIR, XPS, Mössbauer spectroscopy and thermoanalyses. Spectrochim. Acta Part A Mol. Biomol. Spectrosc..

[59] Margenot A.J., Calderón F.J., Goyne K.W., Mukome F.N.D., Parikh S.J. (2017). IR Spectroscopy, Soil Analysis Applications. Encyclopedia of Spectroscopy and Spectrometry.

[60] Simakova Y.S. (2019). Crystal-chemical features of glauconite from Karinskoe deposit (South Urals), Institute of Geology Komi SC UB RAS. Vestn. IG KomiSC.

[61] Povarennykh A.S. (1978). The use of infrared spectra for the determination of minerals. Am. Mineral..

[62] De Aragão J.G.B., Younes M. (2008). Peak separation by derivative spectroscopy applied to FTIR analysis of hydrolized silica. J. Braz. Chem. Soc..

[63] Ferraro J.R., Basile L.J. (2012). Fourier Transform Infrared Spectra: Applications to Chemical Systems.

[64] Holler F., Burns D.H., Callis J.B. (1989). Direct Use of Second Derivatives in Curve-Fitting Procedures. Appl. Spectrosc..

[65] Siqueira L.F.S., Lima K.M.G. (2016). A decade (2004–2014) of FTIR prostate cancer spectroscopy studies: An overview of recent advancements. TrAC Trends Anal. Chem..

[66] Lasch P. (2012). Spectral pre-processing for biomedical vibrational spectroscopy and microspectroscopic imaging. Chemom. Intell. Lab. Syst..

[67] Rinnan Å., van den Berg F., Engelsen S.B. (2009). Review of the most common pre-processing techniques for near-infrared spectra. TrAC Trends Anal. Chem..

[68] Chukanov N.V., Chervonnyi A.D. (2016). Infrared Spectroscopy of Minerals and Related Compounds.

[69] Oloyede O.G., Aroke U.O., Giwa S.O., Jock A.A. (2021). Characterisation of Natural and HDTMA-Br Modified Dijah-Monkin Bentonite Clay: FTIR, XRF, XRD and SEM. Path Sci..

[70] Volkov D., Rogova O., Proskurnin M. (2021). Organic Matter and Mineral Composition of Silicate Soils: FTIR Comparison Study by Photoacoustic, Diffuse Reflectance, and Attenuated Total Reflection Modalities. Agronomy.

[71] So R.T., Blair N.E., Masterson A.L. (2020). Carbonate mineral identification and quantification in sediment matrices using diffuse reflectance infrared Fourier transform spectroscopy. Environ. Chem. Lett..

[72] Shi Z., Sanguinito S., Goodman A., Jessen K., Tsotsis T.T. (2020). Investigation of Mass Transfer and Sorption in CO_2_/Brine/Rock Systems via In Situ FT-IR. Ind. Eng. Chem. Res..

[73] Pärnpuu S., Astover A., Tõnutare T., Penu P., Kauer K. (2022). Soil organic matter qualification with FTIR spectroscopy under different soil types in Estonia. Geoderma Reg..

[74] Stenberg B., Rossel R.A.V., Mouazen A.M., Wetterlind J. (2010). Visible and Near Infrared Spectroscopy in Soil Science. Advances in Agronomy.

[75] Nuzzo A., Buurman P., Cozzolino V., Spaccini R., Piccolo A. (2020). Infrared spectra of soil organic matter under a primary vegetation sequence. Chem. Biol. Technol. Agric..

[76] Krivoshein P.K., Volkov D.S., Rogova O.B., Proskurnin M.A. (2020). FTIR photoacoustic spectroscopy for identification and assessment of soil components: Chernozems and their size fractions. Photoacoustics.

